# Efficacy of microsurgery for patients with cerebral hemorrhage secondary to gestational hypertension: A systematic review protocol of randomized controlled trial

**DOI:** 10.1097/MD.0000000000017558

**Published:** 2019-10-18

**Authors:** An-bang Wang, Hua Zhang

**Affiliations:** a Department of Neurosurgery, The First People's Hospital of Xianyang; b Department of Gynecology and Obstetrics, Second Affiliated Hospital of Shaanxi University of Chinese Medicine, Xianyang, Shaanxi, China.

**Keywords:** cerebral hemorrhage, efficacy, gestational hypertension, microsurgery, randomized controlled trial, safety

## Abstract

**Background:**

Microsurgery is widely utilized for patients with cerebral hemorrhage (CH). The purpose of this study is to assess the efficacy and safety of microsurgery for the treatment of patients with CH secondary to gestational hypertension (GH).

**Methods:**

Relevant randomized controlled trials in eight electronic databases of Cochrane Library, PUBMED, EMBASE, Web of Science, VIP, WANFANG, Chinese Biomedical Literature Database, and China National Knowledge Infrastructure will be included. All electronic databases will be searched from inceptions to the present without language restriction. RevMan 5.3 software will be applied for statistical analysis.

**Results:**

This study will summarize a high-quality synthesis of maternal mortality, severe maternal complications, maternal quality of life, limbs function, muscle strength, and muscle tone to evaluate the efficacy and safety of microsurgery for patients with CH secondary to GH.

**Conclusions:**

This study will provide evidence that microsurgery is an effective intervention in patients with CH secondary to GH.

**PROSPERO registration number:**

PROSPERO CRD42019145750.

## 1 Introduction

Cerebral hemorrhage (CH) is a very common cerebrovascular disorder in clinical practice.^[1–3]^ Patients who experience such disorder often have difficulty in walking, speaking, and understanding, and paralysis or numbness of the face, arm, or leg, which significantly affect their health-related quality of life.^[4–6]^ Furthermore, it also causes high morbidity and mortality in patients with CH.^[7–9]^ It has been estimated that the mortality rate in such patients can reach 40% to 50%, and 75% of those survivors cannot live independently 1 year post stroke.^[10]^

Many factors can cause CH, especially for pregnant women, such as gestational hypertension (GH).^[5,11,12]^ GH is a major predictor of pregnancy-associated CH.^[13,14]^ Several managements are responsible for patients with CH secondary to the GH, including microsurgery, medication, acupuncture, moxibustion, and Tuina, especially for microsurgery.^[15–22]^ A variety of studies have reported that microsurgery is an effective management for patients with CH secondary to the GH.^[4,22–24]^ However, convinced evidence-based level is still needed to support this therapy. Therefore, this study aims to summarize and critically assess the evidence from current clinical studies that have investigated the efficacy of microsurgery as a treatment for patients with CH secondary to the GH.

## 2 Methods

### 2.1 Study selection criteria

#### 2.1.1 Types of studies

We will consider randomized controlled trials (RCTs) comparing microsurgery with other treatment. However, case studies, case-control studies, reviews, and non-RCTs will be excluded.

#### 2.1.2 Types of interventions

The participants in the experimental group must receive microsurgery for CH secondary to GH.

The participants in the control group can receive any treatments, except microsurgery.

#### 2.1.3 Types of participants

Pregnant participants of any age with clinically diagnosed as CH secondary to GH will be considered for inclusion.

#### 2.1.4 Types of outcome measurements

The outcomes comprise of maternal mortality, severe maternal complications (such as number of participants with pre-eclampsia, pre-term labor, and pregnancy loss), maternal quality of life (as measured by Maternal Perceived Quality of Life, or relevant scales), limbs function, muscle strength, and muscle tone.

### 2.2 Search strategy

The electronic databases of Cochrane Library, PUBMED, EMBASE, Web of Science, VIP, WANFANG, Chinese Biomedical Literature Database, and China National Knowledge Infrastructure will be searched from inceptions to the present without language limitation. The detailed strategy for searching the Cochrane Library will be shown in Table 1. Similar detailed search strategies will be modified and adapted to other electronic databases.

**Table 1:**
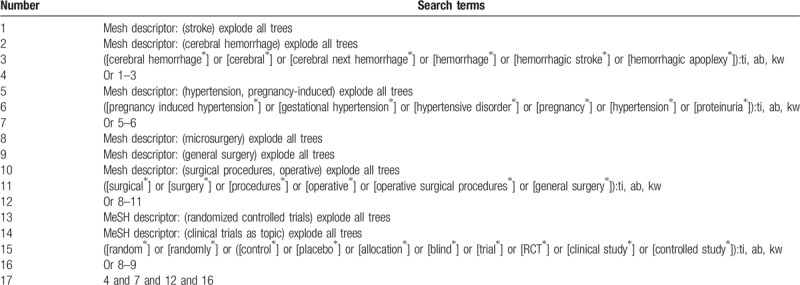
Search strategy for Cochrane Library.

In addition, clinical trials registry, conference proceedings, dissertations, and reference lists of included studies will be searched.

### 2.3 Data collection and analysis

#### 2.3.1 Study selection

Two authors will independently scan the titles and abstracts of all literature identified, and all irrelevant records and duplicated studies will be excluded. The remaining studies will be subsequently screened by reading full-text. In the event of divergences between two authors that they can not solve via discussion, and a third author will be consulted, who can help make the decision. The whole process of study selection will be presented in flowchart.

#### 2.3.2 Data extraction

Data from the eligible studies will be extracted and collected by two authors independently according to the previous designed data extraction sheet. Any disagreements will be resolved by consensus with the help of a third author. We will collect information, such as title, first author, publication year, characteristics of study and patients, study methods, treatment details, outcome indicators, adverse events, and follow-up details. We will contact primary authors of trials for further information when necessary.

#### 2.3.3 Missing data dealing with

We will try to require information by contacting corresponding author of the primary studies for the missing, unclear or insufficient data. If we cannot obtain these data, only available data will be analyzed based on the intent-to-treat principle.

#### 2.3.4 Risk of bias assessment

The risk of bias will be assessed by two independent authors using Cochrane risk of bias tool. It has 7 dimensions, and each one is further classified as high, unclear and low risk of bias. Any different opinions between two authors will be resolved by discussion with the help of a third author.

#### 2.3.5 Measures of treatment effect

Enumeration outcome data will be evaluated as risk ratio and 95% confidence intervals, and continuous outcome data will be calculated as mean difference or standardized mean difference and 95% confidence intervals.

#### 2.3.6 Assessment of heterogeneity

The heterogeneity among eligible studies will be identified using *I*^2^ statistics. *I*^2^ ≤ 50% indicates minor heterogeneity, while *I*^2^ > 50% means substantial heterogeneity.

#### 2.3.7 Assessment of reporting bias

When more than 10 eligible RCTs are included, funnel plot and Egger's regression test will be conducted for reporting bias identification.^[25,26]^

#### 2.3.8 Subgroup analysis

We will carry out subgroup analysis to investigate the potential factors of significant heterogeneity based on the different study characteristics, treatments and controls, and outcome measurements.

#### 2.3.9 Sensitivity analysis

When sufficient studies are available, we will conduct sensitivity analysis to check the stability of outcome results by removing low quality studies.

#### 2.3.10 Ethics and dissemination

Ethical approval will not be required because this study will not use individual patient data. The results of this study are expected to disseminate by the publication in a peer-reviewed journal or conference proceedings.

### 2.4 Data synthesis

RevMan 5.3 software will be adopted for statistical analysis. Meta-analysis will be performed if more than two or above eligible studies with minor heterogeneity at same outcome measurements will be included. If there is minor heterogeneity among sufficient studies (*I*^2^ ≤ 50%), a fixed-effect model will be used for data synthesizing, and meta-analysis will be employed. If there is significant heterogeneity among enough studies (*I*^2^ > 50%), the source of such substantial heterogeneity will be further analyzed, a random-effect model will be used for data pooling, and subgroup analysis will be carried out. If there is still substantial heterogeneity after subgroup analysis, we will not conduct data pooling and meta-analysis. At the same time, outcome results will be reported as narrative summary.

## 3 Discussion

Although previous studies have reported that microsurgery is utilized for the treatment of patients with CH secondary to GH, its efficacy and safety has not been assessed systematically. Thus, it is crucial to make sure whether microsurgery is a good option for the patients with CH secondary to GH. The objective of this study is to systematically assess the efficacy and safety of microsurgery for the treatment of patients with CH secondary to GH. We hope this review will provide most recent information on the credibility current evidence and research directions for both clinical practice and future studies.

## Acknowledgments

This study is supported by Shaanxi Science and Technology Department Science and Technology Development Plan Project (2014K11-02-04-09). The supporter was not allowed to participate this study.

## Author contributions

**Conceptualization:** An-bang Wang, Hua Zhang.

**Data curation:** An-bang Wang, Hua Zhang.

**Formal analysis:** An-bang Wang.

**Investigation:** Hua Zhang.

**Methodology:** An-bang Wang, Hua Zhang.

**Project administration:** Hua Zhang.

**Resources:** An-bang Wang, Hua Zhang.

**Software:** An-bang Wang.

**Supervision:** Hua Zhang.

**Validation:** An-bang Wang, Hua Zhang.

**Visualization:** An-bang Wang, Hua Zhang.

**Writing – original draft:** An-bang Wang, Hua Zhang.

**Writing – review & editing:** An-bang Wang, Hua Zhang.

## References

[1] Suzuki K Sakamoto T Clinical epidemiology of cerebral hemorrhage. Nihon Rinsho 2006;64:315 9. 17469571

[2] Ueno M Hypertensive cerebral hemorrhage. Nihon Rinsho 2004;62:363 8. 15171400

[3] Wilterdink JL Feldmann E Cerebral hemorrhage. Adv Neurol 1994;64:13 23. 8291461

[4] Nakase H Motoyama Y Yamada S Cerebral hemorrhage. Nihon Rinsho 2016;74:671 6. 27333758

[5] Zubkov AY Wijdicks EF Deep venous thrombosis prophylaxis in cerebral hemorrhage. Rev Neurol Dis 2009;6:21 5. 19367220

[6] González-Duarte A Cantú C Ruíz-Sandoval JL Recurrent primary cerebral hemorrhage: frequency, mechanisms, and prognosis. Stroke 1998;29:1802 5. 9731598 10.1161/01.str.29.9.1802

[7] Escudero Augusto D Marqués Alvarez L Taboada Costa F Up-date in spontaneous cerebral hemorrhage. Med Intensiva 2008;32:282 95. 18601836 10.1016/s0210-5691(08)70956-2

[8] Ye L Gao L Cheng H Inflammatory profiles of the interleukin family and network in cerebral hemorrhage. Cell Mol Neurobiol 2018;38:1321 33. 30027390 10.1007/s10571-018-0601-x PMC11481843

[9] Proust F Leveque S Derrey S Spontaneous supratentorial cerebral hemorrhage: role of surgical treatment. Neurochirurgie 2007;53 (2-3 Pt 1):58 65. 17445841 10.1016/j.neuchi.2006.12.003

[10] Klebe D McBride D Flores JJ Modulating the immune response towards a neuroregenerative Peri-injury Milieu after cerebral hemorrhage. J Neuroimmune Pharmacol 2015;10:576 86. 25946986 10.1007/s11481-015-9613-1 PMC4636976

[11] Zou Y Zhang W Huang C Clinical significance of neutrophil to lymphocyte ratio and platelet to lymphocyte ratio in acute cerebral hemorrhage with gastrointestinal hemorrhage, and logistic regression analysis of risk factors. Exp Ther Med 2019;18:1533 8. 31410106 10.3892/etm.2019.7778 PMC6676203

[12] Tzourio C Arima H Harrap S APOE genotype, ethnicity, and the risk of cerebral hemorrhage. Neurology 2008;70:1322 8. 18256366 10.1212/01.wnl.0000308819.43401.87

[13] Say L Chou D Gemmill A Global causes of maternal death: a WHO systematic analysis. Lancet Global Health 2014;2:e323 33. 25103301 10.1016/S2214-109X(14)70227-X

[14] Khan KS Wojdyla D Say L WHO analysis of causes of maternal death: a systematic review. Lancet (London, England) 2006;367:1066 74. 10.1016/S0140-6736(06)68397-9 16581405

[15] Yuan P Bao CL Dong GR Clinical safety research of penetrating acupuncture at the head points for cerebral hemorrhage at the acute stage. Zhongguo Zhen Jiu 2012;32:577 81. 22997780

[16] Song GF Wu CJ Dong SX Rehabilitation training combined acupuncture for limb hemiplegia caused by cerebral hemorrhage: a protocol for a systematic review of randomized controlled trial. Medicine (Baltimore) 2019;98:e14726. 30817621 10.1097/MD.0000000000014726 PMC6831227

[17] Dong XS Song GF Wu CJ Effectiveness of rehabilitation training combined with acupuncture on aphasia after cerebral hemorrhage: a systematic review protocol of randomized controlled trial. Medicine (Baltimore) 2019;98:e16006. 31192945 10.1097/MD.0000000000016006 PMC6587597

[18] Xia ZY Wang J Guo JW Effect of Chinese drugs for breaking blood expelling stasis on acute cerebral hemorrhage: a prospective randomized double-blind controlled study. Zhongguo Zhong Xi Yi Jie He Za Zhi 2016;36:821 6. 30634209

[19] Hu W Xin Y Chen X Tranexamic acid in cerebral hemorrhage: a meta-analysis and systematic review. CNS Drugs 2019;33:327 36. 30741383 10.1007/s40263-019-00608-4

[20] Mizutani T Kojima H Miki Y Arterial dissections of penetrating cerebral arteries causing hypertension-induced cerebral hemorrhage. J Neurosurg 2000;93:859 62. 11059669 10.3171/jns.2000.93.5.0859

[21] Mao Y Nan GX Zhang L Contrast extravasation mimics cerebral hemorrhage in acute ischemic stroke after Solitaire FR clot retrieval and intraarterial thrombolysis: a case report. Acta Neurol Belg 2015;115:723 5. 25944512 10.1007/s13760-015-0481-5

[22] Negură Marderos A Aldea MJ Buzea T Cerebral hemorrhage occurring in pregnancy. Rev Med Chir Soc Med Nat Iasi 1976;80:39 43. 968270

[23] Guo R Blacker DJ Wang X Practice patterns for neurosurgical utilization and outcome in acute intracerebral hemorrhage: intensive blood pressure reduction in acute cerebral hemorrhage trials 1 and 2 Studies. Neurosurgery 2017;81:980 5. 28605557 10.1093/neuros/nyx129

[24] Jia Y Zhong X Liu J Clinical study on Zhuyu Xiaozhong mixture combined with stereotaxic drainage in treating hypertensive cerebral hemorrhage. Zhongguo Zhong Xi Yi Jie He Za Zhi 2000;20:498 500. 11789204

[25] Sutton AJ Duval SJ Tweedie RL Empirical assessment of effect of publication bias on meta-analyses. BMJ 2000;320:1574 7. 10845965 10.1136/bmj.320.7249.1574 PMC27401

[26] Egger M Davey Smith G Schneider M Bias in meta-analysis detected by a simple, graphical test. BMJ 1997;315:629 34. 9310563 10.1136/bmj.315.7109.629 PMC2127453

